# Relief Effects of Icariin on Inflammation-Induced Decrease of Tight Junctions in Intestinal Epithelial Cells

**DOI:** 10.3389/fphar.2022.903762

**Published:** 2022-06-08

**Authors:** Yanli Li, Jie Liu, Pawin Pongkorpsakol, Zhengguo Xiong, Li Li, Xuemei Jiang, Haixia Zhao, Ding Yuan, Changcheng Zhang, Yuhui Guo, Yaoyan Dun

**Affiliations:** ^1^ Third-grade Pharmacological Laboratory on Traditional Chinese Medicine, State Administration of Traditional Chinese Medicine, Medical College, China Three Gorges University, Yichang, China; ^2^ Department of Medical Research Center, Xi’an No. 3 Hospital, The Affiliated Hospital of Northwest University, Xi’an, China; ^3^ Princess Srisavangavadhana College of Medicine, Chulabhorn Royal Academy, Bangkok, Thailand; ^4^ Department of Anatomy and Histoembryology, Medical College, China Three Gorges University, Yichang, China; ^5^ Department of Pathology, Medical College, China Three Gorges University, Yichang, China; ^6^ Department of Traditional Chinese Medicine, Medical College, China Three Gorges University, Yichang, China

**Keywords:** ICA, inflammatory cytokines, tight junction, intestinal barrier function, miR-122a

## Abstract

Inflammatory cytokines including TNF-α and IL-1β impair intestinal barrier function in aging by disrupting intestinal tight junction integrity. Icariin (ICA) has a variety of pharmacological effects. Indeed, ICA produces anti-inflammatory, anti-oxidative stress, and inhibitory effects on microRNA (miRNA) expression. This study was to explore whether ICA could alleviate inflammation-associated intestinal barrier function impairment in aging and its underlying mechanism. Of particular interest, network pharmacology prediction indicated the potential therapeutic impacts of ICA for the treatment of colitis. Then, rats were used to study whether ICA has a protective effect on the reduction of tight junctions caused by inflammatory cytokines. Next, Caco-2 cell monolayers were used to explore the mechanism by which ICA alleviates the down-regulation of tight junctions. Network pharmacology prediction revealed that ICA alleviated colitis *via* suppressing oxidative stress. After ICA intervention, expressions of inflammatory cytokines were reduced, but tight junctions, antioxidant enzymes in aging rats were up-regulated. ICA reversed the TNF-α-induced decrease in abundance of Occludin protein in Caco-2 cell monolayers. Meanwhile, ICA alleviated the increase in permeability and expression of miR-122a. However, the protective effect of ICA was markedly attenuated after transfection with miR-122a mimics. In conclusion, ICA reduced the expressions of Occludin, Claudin1, and Claudin5 in colon, which were related to the reduction of TNF-α and IL-1β and alleviation of colonic in *vivore*. And ICA attenuated TNF-α-induced Occludin disruption and epithelial barrier impairment by decreasing miR-122a expression in Caco-2 cell monolayers.

## Introduction

The intestinal lumen contains a complex microenvironment including a large number of commensal and pathogenic bacteria, toxoids, dietary antigens, and is generally exposes to extreme changes of pH, etc (Xu et al., 2011; Zhao et al., 2020). The intestinal mucosal barrier separates body’s internal milieu from the intestinal contents. This tissue barrier protects against mucosal-to-serosal permeability of harmful substances from intestinal lumen to blood circulation. Impairment of the intestinal barrier function leads to pathogenesis of several diseases including diarrhea, intestinal irritability syndrome, gastrointestinal tumor development, steatohepatitis, viral hepatitis, etc (Chen et al., 2015; Zeisel et al., 2019). The most important role of intestinal barrier is the mechanical barrier composed of intestinal epithelial cells and tight junctions between cells. Tight junction can regulate paracellular permeability by controlling the size of the intercellular space (König et al., 2016). Occludin is the core component of the tight junction protein complex, and down-regulated expression of Occludin markedly increases intestinal permeability (Turner et al., 2014; Hou et al., 2017). In addition, the tight junction Claudins, such as Claudin1, Claudin5, etc, together with Occludin constitute the main skeleton of the tight junction (Awad et al., 2017). In H_2_O_2_-induced disruption of Occludin and ZO-1 in Caco-2 cells and rat intestinal epithelium, intestinal barrier function was impaired, conversely, improving the expression of tight junctions alleviated intestinal barrier disruption (Wang et al., 2016). Thus, the integrity of tight junctions is important for maintaining intestinal barrier function. Therefore, mechanisms and interventions of tight junction disruption deserve attention.

Several lines of evidence have revealed that a variety of inflammatory cytokines can down-regulate the expressions of tight junctions. Indeed, TNF-α, which is mainly derived from T lymphocytes and macrophages, plays an important role in the decrease of Occludin. Previous studies have shown that TNF-α attenuated the epithelial barrier function by decreasing Occludin expression in the intestine of mice (Watari et al., 2017). On the other hand, the specific overexpression of Occludin in intestinal epithelial cells limits the barrier loss induced by TNF-α and prevents the occurrence of diarrhea (Marchiando et al., 2010). Other inflammatory cytokines can also disrupt intestinal barrier function, for example, IL-1β interferes the integrity of Occludin, Claudin1, and Claudin7 in Caco-2 cells (Wang et al., 2017). Increased levels of intestinal inflammatory cytokines IL-1β, interferon-γ and IL-6, and down-regulated tight junction protein expression in elderly baboons led to intestinal mucosal barrier disorders (Tran and Greenwood-Van Meerveld, 2013). Concurrently, we also found that the expressions of Occludin and Claudin-1 in the colon of aging rats were decreased, and the expressions of TNF-α and IL-1β were increased (Dun et al., 2018). Mabbott NA et al. further examined the human gut and found that increased intestinal permeability in the elderly is accompanied by enhanced levels of pro-inflammatory cytokines (Mabbott*.*, 2015), suggesting that up-regulated expression of inflammatory cytokines can impair the barrier function by disrupting tight junctions during aging. However, the specific molecular mechanism by which inflammatory cytokines regulate tight junctions remains unclear.

Recent studies showed that miRNAs were critical in the down-regulation of Occludin protein caused by inflammatory cytokines. Because Occludin mRNA has a long 3’untranslated region, it is feasible to become recognized by miRNA. Studies in mice and Caco-2 cells have shown that TNF-α significantly induced the expression of intestinal epithelial miR-122a, which was able to bind to the 3’untranslated region of Occludin, leading to degradation of Occludin mRNA and protein and defective intestinal mucosal barrier function (Ye et al., 2011). In addition, miR-200c, miR-429, miR-144 could directly target mouse intestinal epithelial Occludin mRNA stability (Hou et al., 2017; Rawat et al., 2020).

ICA, the main active ingredient of Epimedium, is a natural flavonoid compound extracted from Epimedium of the Berberis family. ICA plays a potential role in up-regulating the level of intestinal Occludin protein and alleviating intestinal barrier dysfunction. ICA alleviated intestinal inflammation in mouse model of Dextran sulfate sodium-induced colitis (Tao et al., 2013), and could also reverse attenuated expression of Occludin protein and the impaired barrier function in the porcine jejunum caused by *E. coli*, and hence reduced diarrhea (Xiong et al., 2020). Current studies have also found that ICA inhibited the expression of miR-34C, miR-625-3p, and miR-335 (Liu et al., 2016; Fang et al., 2019; Yue et al., 2021). The above studies indicated that ICA could exert its pharmacological effects by regulating a variety of miRNAs. Therefore, we hypothesized that ICA could have a protective effect on tight junction damage caused by inflammatory cytokines in the epithelium, at least in part, by the down-regulation of related miRNAs.

## Materials and Methods

### Analysis of ICA Action Target

The 2D structure of the ICA was retrieved by Pubchem. The 2D structure of ICA was input into Swiss Target Prediction to predict drug targets. Colitis was used to search for disease-related targets in GeneCards and DisGeNet databases. The three groups of predicted targets were intersected and compared by Venn diagram to obtain the potential targets of ICA in the treatment of colitis. The potential targets of ICA in the treatment of colitis were imported into String to analyze the interaction of these targets, and the main targets were screened. The protein interaction network model was obtained by analyzing the targets in Cytoscape3.7.1. The intersection genes in ICA treatment of colitis were imported into the DAVID (Database for Annotation, Visualization, and Integrated Discovery) to perform the KEGG pathway enrichment analysis.

### Experimental Animals and Treatment Methods

A total of sixty Sprague Dawley male rats were purchased from the Experimental Animal Center of China Three Gorges University and raised in the well-ventilated animal room of the Medical College. All experiments were in accordance with the guidelines of the National Institutes of Health on the care and use of animals. The temperature and humidity of the animal room were constantly with a 12-h cycle of light and dark. Two-month-old rats were used as the adult group (*n* = 15), and seventeen-month-old rats were randomly divided into the aging group (*n* = 15), low-dose of ICA (ICA-L) group (*n* = 15), and high-dose of ICA (ICA-H) group (*n* = 15). Rats had food and water *ad libitum*. The rats in the adult and the aging group were fed with a normal diet, and the rats in ICA-L and ICA-H groups were fed with 2 mg/kg and 6 mg/kg ICA diet (Xu et al., 2011; Zhao et al., 2020). All rats were fed for 4 months and then sacrificed. The colonic tissue was quickly divided, and 1.5–2.5 cm of tissue was taken and placed in 4% paraformaldehyde for 24 h, then dehydrated and embedded. The remaining colon tissue was stored in −80°Cfreezer after feces were washed away.

### Colonic Morphology

The embedded colon tissues were sectioned to a thickness of 4 μm. Sections were deparaffinized with xylene and various concentrations of alcohol. Sections were stained with hematoxylin, then differentiated with alcohol containing 1% hydrochloric acid for several seconds, and finally stained with eosin for 2–3 min. The sections were dehydrated and sealed. Changes in colonic morphology were observed by an optical microscope (Olympus, Shanghai, China).

### Immunohistochemistry (IHC)

The tissue sections were deparaffinized and high-pressure antigen repair was performed with citric acid repair solution. After cooling to room temperature, endogenous peroxidase in slices was inactivated with 3% H_2_O_2_ solution. After blocking the non-specific antigen with 5% BSA, primary antibodies were added and placed at 4°C for at least 14 h. Main primary antibodies were Occludin (1:100, Abcam, Cambridge, United Kingdom), Claudin 1 (1:200, Santa Cruz, CA, United States), Claudin 5 (1:200, Zen-Bioscience, Chengdu, China). The corresponding secondary antibody was incubated at room temperature for 1 h. After staining with DAB and hematoxylin, the slices were sealed and placed under an optical microscope (Olympus, Shanghai, China) for observation and image collection. IHC images were analyzed by Image-Pro-Plus7.0 software.

### The Enzyme Activity of Superoxide Dismutase (SOD), Catalase (CAT), and Glutathione Peroxidase (GSH-Px) in the Colon

Colon tissue was homogenized, and the supernatant was collected after centrifugation. The supernatant was used for substrate and enzyme reaction with reference to the kit (Nanjing jiancheng bioengineering institute, Jiangsu, China). The reaction solution was measured with an Infinite M200 PRO multimode reader (Tecan, Switzerland). The data was processed according to the instructions.

### Cell Culture

Caco-2 cells (ATCC, Manassas, VA, United States) were cultured in DMEM medium with 10% fetal bovine serum in an incubator with 5% CO_2_ and 37°C, and then seeded into Transwell chambers (Corning, NY, United States) at a density of 8×10^4^ cells/ml. The pore size of the Transwell membrane was 0.4 µm. AP (apical) side and the BP (basolateral) side of the Transwell chamber were added with culture medium in advance and then placed in an incubator for preheating to improve the adherence efficiency of cells. The culture medium was changed 24 h after the cells were inoculated, the medium was changed every other day for 1 week, after that, and the medium was changed every day for the next 2 weeks. Cells were cultured continuously for 21 days.

### Evaluation of the Establishment of Caco-2 Cell Monolayers

Intestinal alkaline phosphatase (IAP) is a marker enzyme of the brush edge of small intestinal epithelia, which can be used to evaluate the differentiation state of cell (He et al., 2021). At 7, 14, and 21 days after inoculation, the culture medium of AP and BP side was taken, and the IAP activity was measured according to the instructions in the kit (Nanjing jiancheng bioengineering institute, Jiangsu, China).

TEER (Transepithelial Electrical Resistance) of cell monolayers is measured using a transmembrane resistance meter (King Tech, Shenzhen, China). The shorter electrode was placed on the AP side without touching the cells, and the longer electrode was placed on the BP side just touching the bottom, and the measured reading is the resistance value. In addition, a blank chamber without cells was established for each measurement to measure the blank resistance value. TEER= (measured value-blank resistance value) * cell membrane area, the unit is Ω/cm^2^.

### MTT Assay to Determine the Effect of Treatment Factors on the Viability of Caco-2 Cells

Caco-2 cells were seeded in a 96-well plate at a density of 5×10^4^ cells/ml. After culturing for 24 h, MTT was added to each well for further cultivation for 4 h. Then the supernatant was removed. DMSO was added, the orifice plate was placed on the shaking table at a low speed of 10 min, and the OD value of each orifice was measured at the wavelength of 570 nm by the Infinite M200 PRO multimode reader (Tecan, Switzerland). The cell viability of each group was calculated from the percentage of the control group.

### Damage of Cell Monolayer Barrier and Drug Treatment

To establish a monolayer barrier damage model, Caco-2 cell monolayers were treated with medium containing 10 ng/ml or 20 ng/ml TNF-α on the AP side for 24 h or 48 h. Appropriate concentration and time of treatment of TNF-α group monolayers were selected by the expression of tight junctions. ICA was first dissolved in DMSO and then diluted to final concentration through medium. In the ICA group, 5 μM or 50 μM ICA was added to AP side for 0.5 h in advance, then 20 ng/ml TNF-α was added to BP side, and ICA and TNF-α acted together for 24 h. The DMSO group was addressed by adding medium containing 0.1% DMSO on the AP side for 24.5 h.

### Immunofluorescence (IF)

Caco-2 monolayer cells in the Transwell were sucked out of the medium, washed with PBS and fixed with 4% paraformaldehyde. After washing with PBS, cells were permeable by adding 0.1% Triton each. After adding PBS for washing again, each chamber was blocked with 5% BSA in PBS for 1 h. Then, Occludin antibody solution (1:1,000, Abcam, Cambridge, United Kingdom) was prepared and 100 μl was added to each chamber at 4°C overnight. The chamber was washed three times with PBS, and then the corresponding fluorescent secondary antibody was added to incubate for 1 h at room temperature. It is worth noting that from this step it should be strictly guarded against the light. Sections were washed with PBS, incubated with DAPI solution for 5 min at room temperature, and washed with PBS again. The membrane of each chamber was cut with a scalpel, placed on a slide with an anti-fluorescence quencher drop, and a coverslip was placed. Then, the samples were observed and photographed under a laser confocal scanning microscope (Nikon, Tokyo, Japan).

### Western Blot

Caco-2 cell monolayers and colon tissue were extracted with RIPA lysate (Applygen, Beijing, China). The proteins were electrophoresed with 10% SDS-PAGE gel. After electrophoresis, the protein was transferred to the polyvinylidene difluoride membrane and blocked with 5% skim milk at room temperature for 1 h, and then incubated with specific primary antibodies overnight at 4°C. The main primary antibodies were Occludin (1:1,000, Abcam, Cambridge, United Kingdom) and GAPDH (1:5,000, Servicebio, Wuhan, China), β-actin (1:5,000, ABclonal, Wuhan, China), Claudin 1 (1:1,000, Santa Cruz, CA, United States), Claudin 5 (1:2000, Zen-Bioscience, Chengdu, China), TNF-α (1:1,000, Santa Cruz, CA, United States), IL-1β (1:2000, Abcam, Cambridge, United Kingdom), HO-1 (1:1,000, Proteintech, Wuhan, China), NQO-1 (1:1,000, Servicebio, Wuhan, China). After the membrane was washed three times with TBST buffer, it was incubated with the corresponding secondary antibody for 1 h at room temperature. Antibody-bound proteins were detected with ECL electrochemiluminescence reagent. The relative expressions of proteins were analyzed by ImageJ (Rawak Software, Inc. Germany).

### Detection of RNA Expression by Real-Time Quantitative PCR (qPCR)

Total RNA was extracted from cells according to the instruction of RNA Extraction Kit (TAKARA, Beijing, China), and its concentration and OD260/OD280 nm were detected by the nucleic acid tester. cDNA products were synthesized using a reverse transcription (RT) kit (Vazyme, Jiangsu, China). qPCR was carried out using the kit (Vazyme, Jiangsu, China). The relative expression level of target RNA was measured by CT value. Specific primer sequences were shown in Table 1.

**TABLE 1:** This is a table. Reverse transcription (RT).

RNA	Primer
Occludin	Forward: CAT​TGC​CAT​CTT​TGC​ATG​TGT
	Reverse: GGT​AGC​CTA​CAC​TAC​CTC​CTA​TAA
β-actin	Forward: CAC​CCA​GCA​CAA​TGA​AGA​TCA​AGA​T
	Reverse: CCA​GTT​TTT​AAA​TCC​TGA​GTC​AAG​C
U6	Forward: CTCGCTTCGGCAGCACA
	Reverse: AAC​GCT​TCA​CGA​ATT​TGC​GT
miR-122a-5p	RT: GTC​GTA​TCC​AGT​GCA​GGG​TCC​GAG​GTA​TTC​GCA​CTG​GAT​ACG​ACC​AAA CA
	Forward: CGC​GTG​GAG​TGT​GAC​AAT​GG
miR-200c-3p	RT: GTC​GTA​TCG​ACT​GCA​GGG​TCC​GAG​GTA​TTC​GCA​GTC​GAT​ACG​ACT​CCA​TC
	Forward: CGG​CTA​ATA​CTG​CCG​GGT​AA
miR-144-3p	RT: GTC​GTA​TCG​ACT​GCA​GGG​TCC​GAG​GTA​TTC​GCA​GTC​GAT​ACG​ACA​GTA​CA
	Forward: CGG​CCG​GCT​ACA​GTA​TAG​ATG​A
miR-429	RT: GTC​GTA​TCG​ACT​GCA​GGG​TCC​GAG​GTA​TTC​GCA​GTC​GAT​ACG​ACA​CGG​TT
	Forward: GCC​GGC​TAA​TAC​TGT​CTG​GTA​A
miRNA	Common reverse: ACT​GCA​GGG​TCC​GAG​GTA​TT

### Detection of the Permeability of Caco-2 Cell Monolayers

4-KD FITC-dextran is a paracellular permeability tracer, and the flux from AP to BL can reflect the permeability of the cell monolayer (Yu et al., 2016; Chen et al., 2017). After the original medium in Transwell was removed, DMEM medium containing 200 μg/ml 4-KD FITC-dextran was added and placed in an incubator for incubation. After 2 h, the BL side medium was collected and centrifuged to eliminate the precipitate. Fluorescence intensity of 100 μl medium was measured at excitation light 385 nm and emission light 545 nm with an Infinite F200 PRO multimode reader (Tecan, Switzerland). According to the standard curve, the concentration and the transmittance (100%) of 4-KD FITC-dextran (Sigma-Aldrich, Shanghai, China) in the BL side medium were calculated.

### Cell Transfection

To overexpress miR-122a in Caco-2 cells, miR-122a mimics were designed and synthesized by GenePharma (Shanghai, China). Among the RNA sequences, one is miR-122a mimics and the other is an antisense strand designed for stability. After RNA transfection into cells, the antisense strand degrades, leaving a single strand of miR-122a mimics. At the same time, an unrelated nucleotide sequence mimics-negative control (NC) was designed by selecting a mimics RNA template from the full miR-122a mimics sequence. According to the instructions of Lipofectamine 2000(Life Technologies, Carlsbad, United States), Caco-2 cells in culture dish were transfected with the specified dilution volume, and the medium was changed after 24 h. The transfection efficiency was assessed by detecting the expression level of miR-122a. According to the transfection effect, the concentration ratio of Lipofectamine 2000 2.5 μl and RNA 20 pmol was selected to transfect Caco-2 cell monolayers.

### Statistical Analysis

Data were described with mean ± standard error, and analyzed by GraphPad Prism 6.0. One-way analysis of variance was used to compare multiple groups. *p* < 0.05 was considered statistically significant.

## Results

### Results of Network Pharmacology Analysis of ICA

**FIGURE 1:**
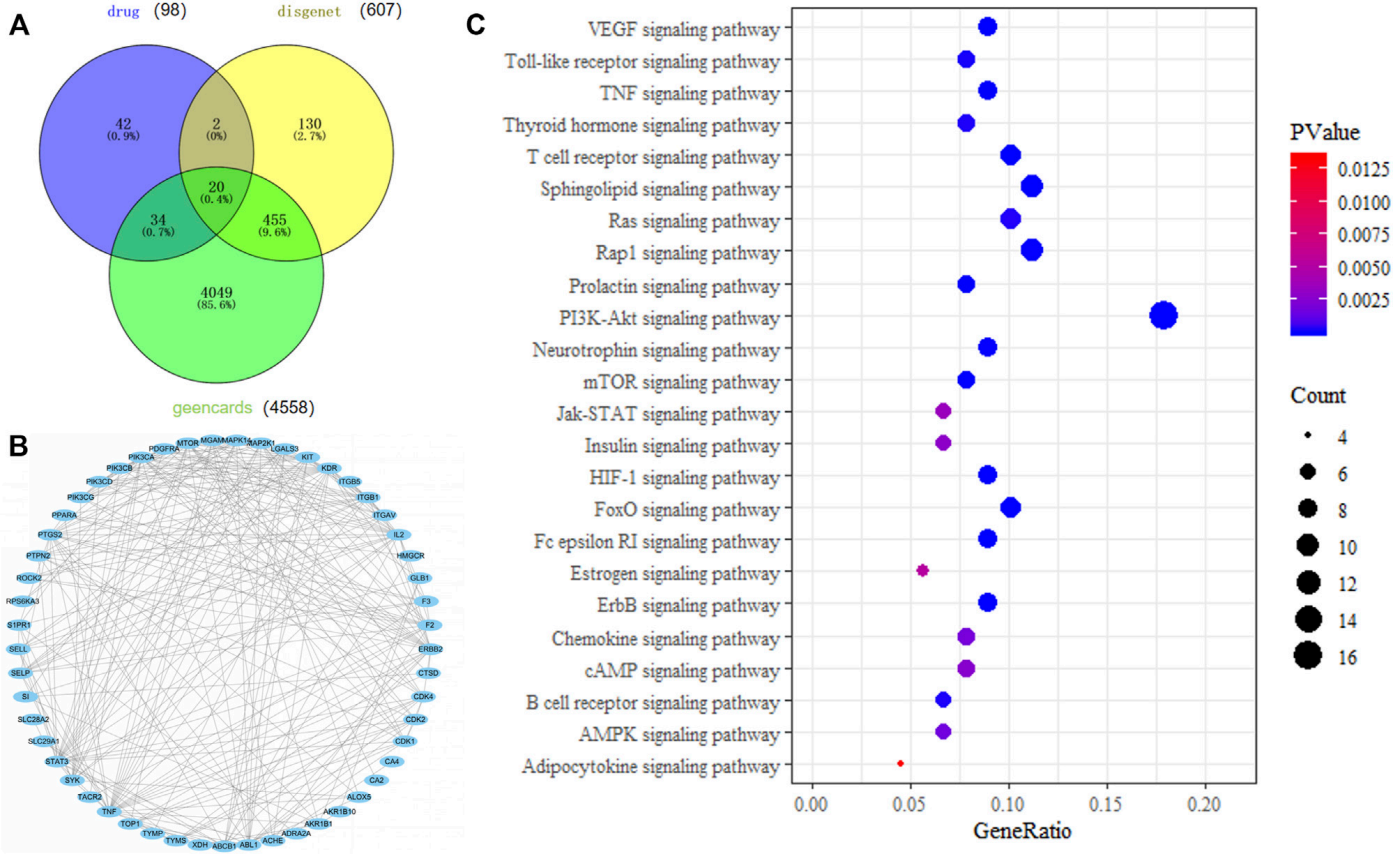
Network pharmacology analysis of the mechanism of action of ICA in the treatment of colitis. **(A)** Venn diagram of intersection of ICA targets and colitis targets. **(B)** ICA target protein interaction network. **(C)** KEGG pathway analysis of ICA target.

Targets of ICA treatment for colitis were analyzed, and the protein interaction network diagram of the related targets was simulated. Intersection targets of the DAVID database were used for KEGG pathway analysis, and a total of 25 KEGG pathways were obtained. As shown in Figure 1A, there were 56 intersection targets between predicted drug targets and disease targets in the Geencard and DisGeNET databases, suggesting that these targets are potential targets for ICA to improve colitis. As shown in Figure 1B, Fifty-three of the 56 potential intersection targets will interact with each other. As shown in Figure 1C, Involvement of ICA and several pathways related to oxidative stress, such as PI3K/AKT signaling pathway, FoxO signaling pathway, HIF-1 signaling pathway, AMPK signaling pathway (Chen et al., 2016; Glover et al., 2016; Sears and Broihier, 2016; Sreekumar and Kannan, 2020; Zhang et al., 2020), suggesting that ICA may reduce oxidative stress, which is good for the treatment of colitis.

### ICA Reduced the Expression of Inflammatory Cytokines TNF-α and IL-1β in the Colon of Aging Rats

**FIGURE 2:**
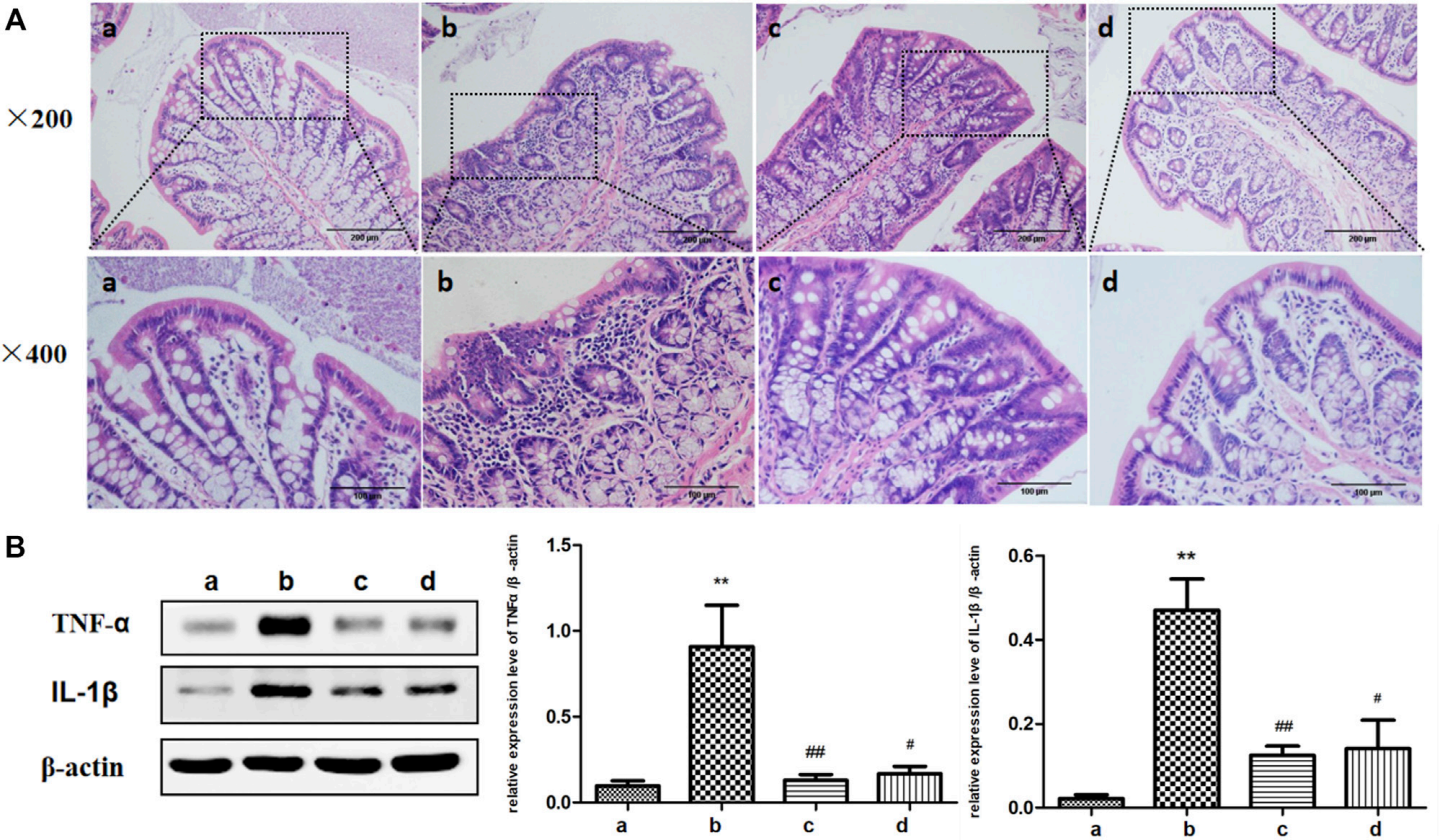
Effects of ICA on colon inflammation in aging rats. **(A)** Representative figures of HE of colonic epithelial morphology (×200, ×400). **(B)** The effect of ICA on the expressions of inflammatory cytokines TNF-α and IL-1β in the colon of aging rats was detected by Western blot. a: Adult group, b: Aging group, c: Low dose of ICA-treated aging group (ICA-L group), d: High dose of ICA-treated aging group (ICA-H group). ^**^ *p* < 0.01 vs. Adult group. ^#^ *p* < 0.05 vs. Aging group. ^##^ *p* < 0.01 vs. Aging group, *n* = 4-8.

Studies showed that Colitis could occur during the process of aging (Tran and Greenwood-Van Meerveld, 2013) and ICA had significant anti-inflammatory effects (Kong et al., 2015). Therefore, the effect of ICA on colonic inflammation in aging was examined. HE staining was used to observe the morphological changes of colonic epithelium and intestinal inflammation levels were detected in each group. As shown in Figure 2A, the colonic epithelial cells of the rats in the young group were neatly arranged. In the aging group, a large number of inflammatory cells were infiltrated in the colonic mucosa and the arrangement of colonic epithelial cells was abnormal, which was relieved by the treatment of ICA. The development of tissue inflammatory response was closely related to the expression of proinflammatory cytokines TNF-α and IL-1β. As shown in Figure 2B, compared with those in the young rats, the protein expressions of TNF-α and IL-1β in the colon tissue of the aging rats were upregulated, which could be reversed by treatment with ICA. The results showed that ICA could improve colitis in aging rats.

### ICA Increased the Expression of Tight Junction Protein in the Colonic Epithelium of Aging Rats

**FIGURE 3:**
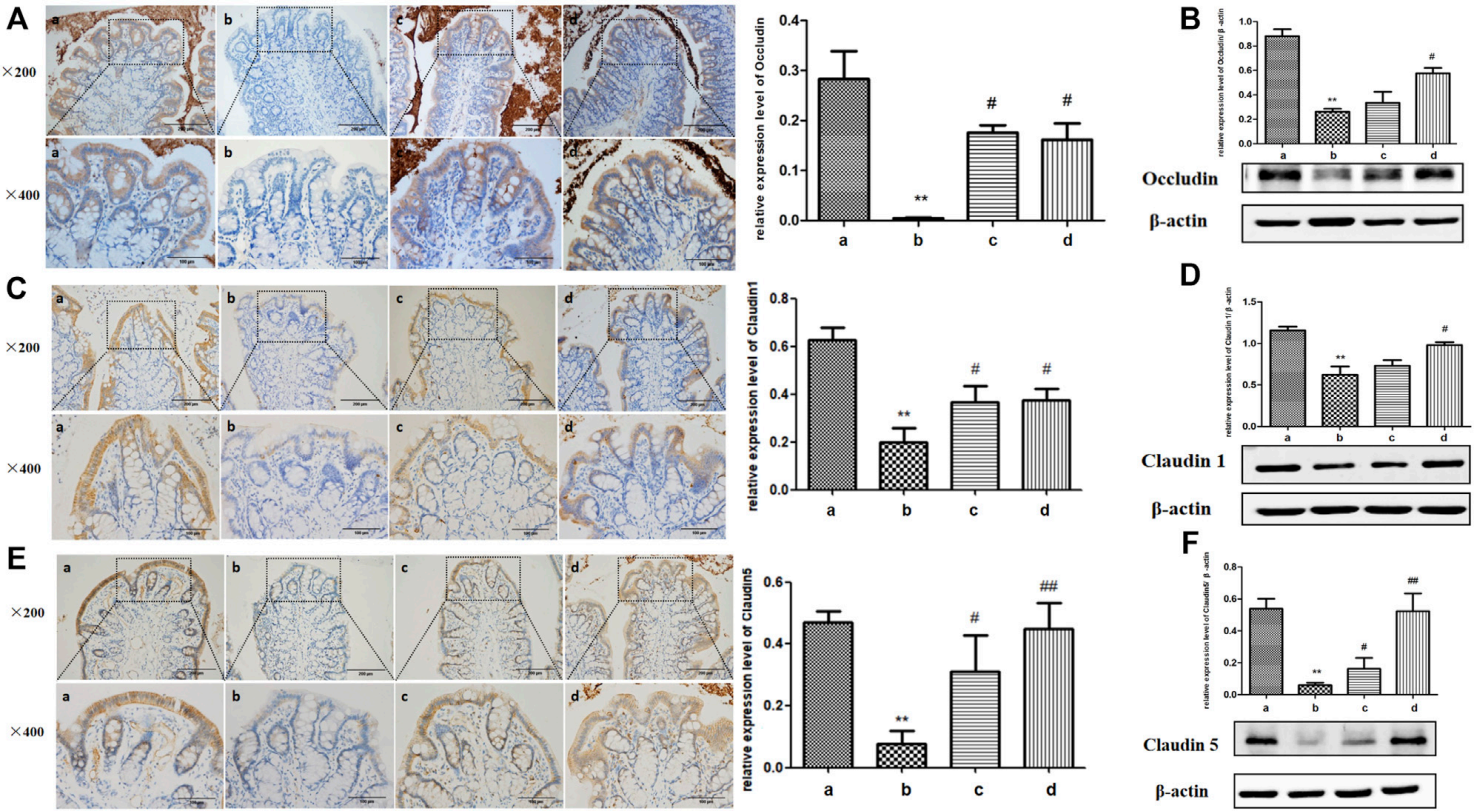
Effects of ICA on tight junction proteins of colonic epithelium in aging rats. Expressions of **(A,B)** Occludin, **(C,D)** Claudin-1, and **(E,F)** Claudin5 between colonic epithelial cells in aging rats were detected by IHC and Western blot (×200, ×400). a: Adult group, b: Aging group, c: ICA-L group, d: ICA-H group. ^**^ *p* < 0.01 vs. Adult group. ^#^ *p* < 0.05 vs. Aging group. ^##^ *p* < 0.01 vs. Aging group, *n* = 3-4.

Tight junctions, are composed of a dense network of tight junction proteins such as Occludin, Claudin-1, and Claudin-5, which are gradually decreased under the stimulation of inflammatory cytokines (Yang et al., 2017). Therefore, the expressions of tight junction proteins were detected by IHC and Western blot. As shown in Figure 3, tight junction proteins were mainly distributed between the intestinal epithelial cells. The expressions of Occludin, Claudin-1 and Claudin-5 in the intestinal tissues of young rats were obvious, while those in the aging rats were markedly impaired. Tight junction protein expressions were almost promoted in both the ICA-L and ICA-H groups in aging rats. Therefore, ICA mitigated the damage of colonic tight junction protein caused by inflammatory cytokines during the process of aging in rats.

### ICA Reduced Colonic Oxidative Stress in Aging Rats

**FIGURE 4:**
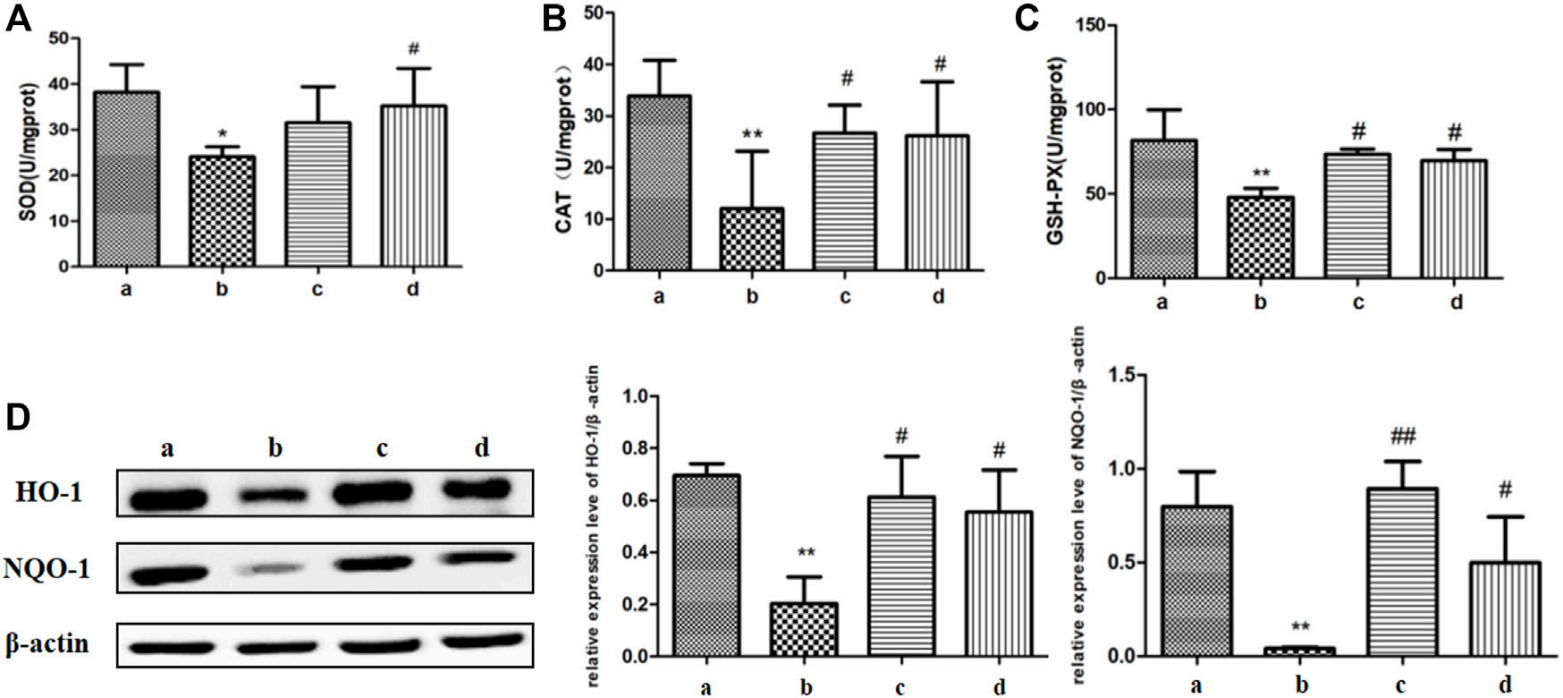
Effects of ICA on colonic oxidative stress in aging rats. The regulation of ICA on antioxidant enzymes **(A)** SOD, **(B)**CAT, and **(C)**GSH-Px in the colon of aging rats were detected by biochemical kits. **(D)** The expressions of HO-1 and NQO-1 in the colon of aging rats were detected by Western blot. a: Adult group, b: Aging group, c: ICA-L group, d: ICA-H group. ^*^ *p* < 0.05 vs. Adult group. ^**^ *p* < 0.01 vs. Adult group. ^#^ *p* < 0.05 vs. Aging group. ^##^ *p* < 0.01 vs. Aging group, *n* = 5-8.

The imbalance of oxidative and antioxidant systems in aging organisms leads to the accumulation of reactive oxygen species (ROS) in the body and causes oxidative damage to tissues, resulting in down-regulation of tight junctions (Diaz de Barboza et al., 2017). Therefore, we determined some antioxidant-related proteases, which include SOD, CAT, and GSH-Px in the colon of aging versus young rats. As shown in Figure 4, compared with the youth rats, the SOD enzyme activity level in the colon tissue of the aging group was suppressed, and the CAT enzyme and GSH-Px enzyme activity levels were also decreased. Compared with the aging group, the activity levels of CAT enzyme and GSH-Px enzyme in the colon of aging rats in the ICA-L group were significantly increased; the activity levels of SOD enzyme, CAT enzyme, and GSH-Px enzyme in the ICA-H group were up-regulated.

Both heme oxygenase 1 (HO-1) and NADPH Quinone acceptor Oxidoreductase 1 (NQO-1) can alleviate oxidative stress by eliminating excess ROS (Li et al., 2018). Compared with the young rats, the expression of HO-1 and NQO-1 proteins in colon tissues of the rats in the aging group was obviously impaired, which was improved markedly in both ICA-L and ICA-H groups. The results showed that ICA alleviated oxidative stress in the colon of aging rats by elevating the activities of antioxidant enzymes.

### The Increased Concentration of TNF-α Resulted in the Decrease of Occludin in Caco-2 Cell Monolayers

**FIGURE 5:**
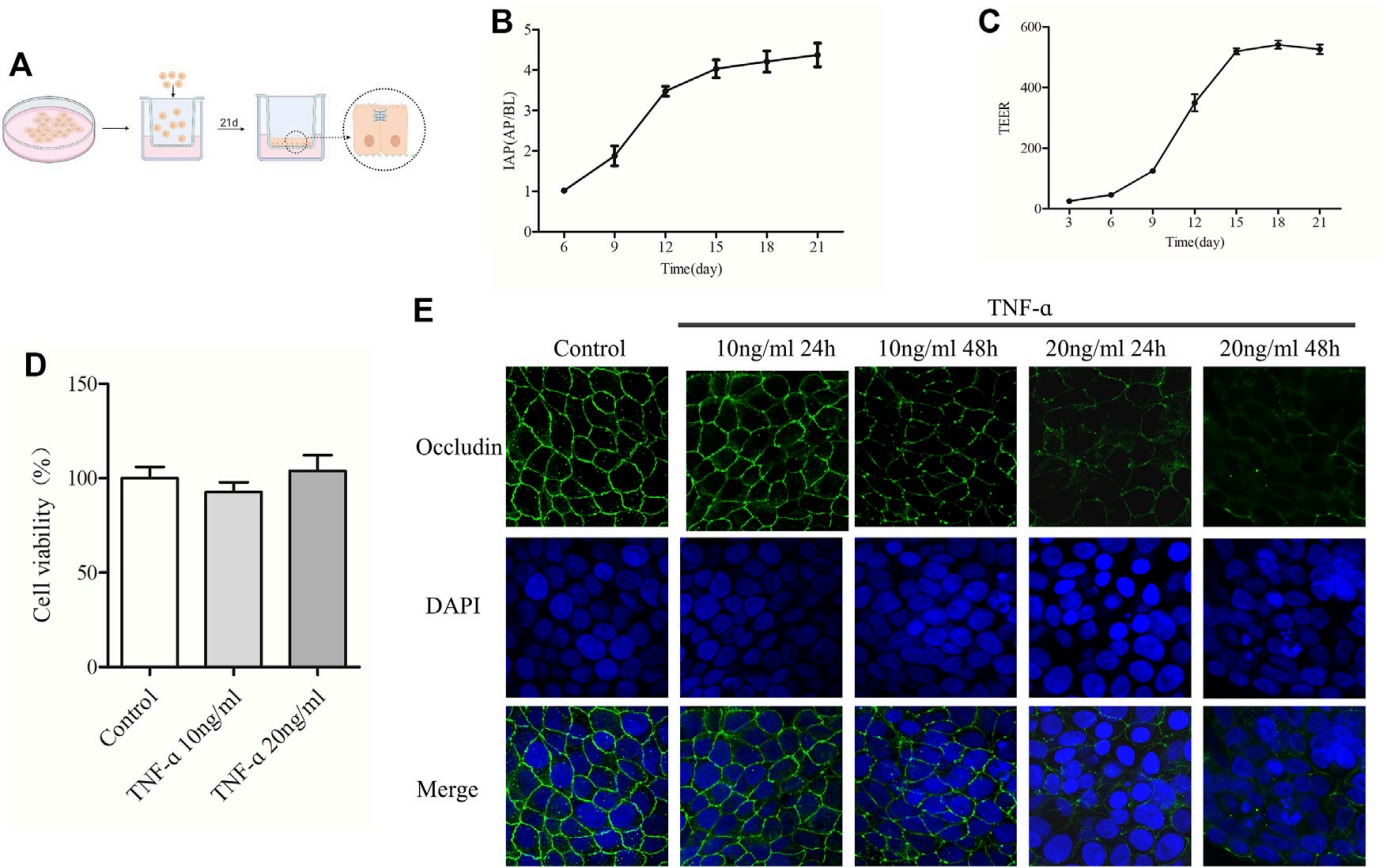
The increased concentration of TNF-α resulted in the decrease of Occludin in Caco-2 cell monolayers. **(A)** Schematic diagram of culturing Caco-2 cell monolayers in the Transwell. **(B)** IAP activity ratio of AP and BP in different days of culture was detected. **(C)** TEER value of Caco-2 cell monolayers was detected on different days. **(D)** MTT was used to detect the effect of TNF-α at 10 ng/ml or 20 ng/ml on caco-2 cell viability. **(E)** Caco-2 cell monolayers were treated with 10 ng/ml or 20 ng/ml TNF-α for 24 or 48 h, and the expression of Occludin was detected by IF, *n* = 3–10.

The above study showed that ICA eased the damage of colonic tight junctions in aging rats, and the potential mechanism was related to the reduction of inflammatory cytokines and colonic oxidative stress. The specific mechanism by which ICA alleviates the damage of tight junctions caused by inflammatory cytokines was further studied *in vitro*. Caco-2 cells are human colon cancer cells, which are widely used in the study of the barrier function in intestinal epithelial monolayer because of their human-relevant intestinal structure and function (Rawat et al., 2020). As shown in Figure 5A, Caco-2 cells were seeded in a Transwell chamber and cultured continuously for 21 days. During the differentiation process of Caco-2 cells, the cells continuously secrete the marker enzyme-IAP to the AP side. When the ratio of IAP activity in the AP side and BP side is over 3, indicating that the monolayer barrier is relatively complete (He et al., 2021). TEER is one of the best methods for assessing tight junction integrity in the Caco-2 cell monolayers model. TEER represents intestinal permeability. It is generally accepted that TEER >330 Ω/cm^2^ indicates the formation of an intact cell monolayer and tight junctions (Stevens et al., 2022). Therefore, measurement of IAP activity ratio on the AP and BL sides and TEER can be used to assess whether the intestinal epithelial model is established. As shown in Figures 5B,C, the IAP ratio and TEER gradually increased with the extension of culture time. On the 21st day of culture, the IAP ratio reached 4.5, and the TEER reached around 500 Ω/cm^2^. The above results indicated that the cells cultured for 21 days had formed a complete and successive monolayer, and an *in vitro* monolayer cell model was established.

To select the appropriate TNF-α concentration, cell viability and the expression of Occludin in TNF-α-induced Caco-2 cell monolayers were measured. As shown in Figure 5D, treatment with TNF-α at a concentration of 10 ng/ml or 20 ng/ml for 24 h had no significant effect on the viability of Caco-2 cells. Figure 5E showed that the Occludin protein of Caco-2 cell monolayers in the control group was expressed along the cell membrane, which was polygonal linear, with clear and continuous edges, and outlined the typical paving stone-like structure of epithelial cells. Indeed, there was no obvious gap between the cells. After treatment with 10 ng/ml TNF-α for 24 h, the expression of Occludin showed no significant change, and after treatment for 48 h, the linear fluorescence of Occludin was broken, and the signal was weakened. When treated with 20 ng/ml TNF-α for 24 and 48 h, the fluorescence signal of Occludin protein was visibly weakened, the fluorescence band was broken, and obvious gaps appeared between cells. Degradation of Occludin was enhanced with time at 20 ng/ml TNF-α. According to the results of IF and MTT, the model group selected 20 ng/ml TNF-α for 24 h in the following experiments.

### ICA Attenuated Occludin Decrease of Caco-2 Cell Monolayers Induced by TNF-α

**FIGURE 6:**
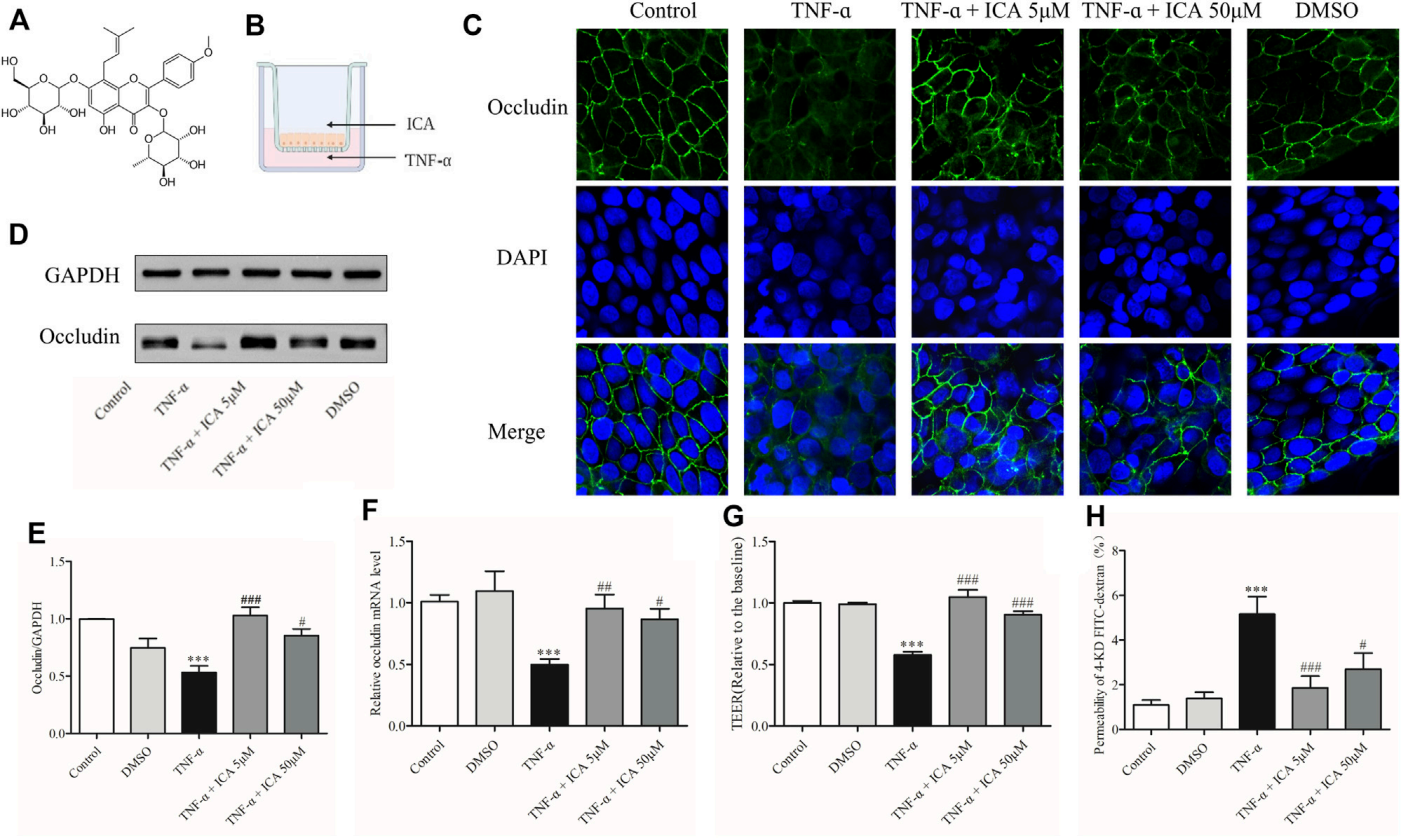
ICA reversed the decrease in Occludin expression and barrier degradation of Caco-2 cell monolayers caused by TNF-α stimulation. **(A)** The structure of ICA. **(B)** Patterns of administration of TNF-α and ICA. **(C)** The changes of Occludin were detected by IF (800×). In the ICA group, 5 μM ICA or 50 μM ICA was added to the AP side for 30 min, and 20 ng/ml TNF-α was added to the BP side for co-treatment for 24 h. In the DMSO group, 1 μl/ml DMSO was added to the AP side then treatment for 24.5 h. The expression of Occludin protein **(D,E)** and mRNA **(F)** was detected by Western blot or qPCR. **(G)**TEER and **(H)** 4-KD FITC-dextran detected changes in barrier permeability of monolayers. ^***^ *p* < 0.001 vs. Control group. ^#^ *p* < 0.05 vs. TNF-α group. ^##^ *p* < 0.01 vs. TNF-α group. ^###^ *p* < 0.001 vs. TNF-α group *n* = 8–27.

Figure 6A is the structure of ICA. According to the previous evidence, the effective ICA concentration was indicated as 5 and 50 μM (Chen et al., 2019; Xiong et al., 2020). As shown in Figure 5B ICA was added to the AP side fand TNF-α was added to the BP side for co-treatment. As shown in Figure 6C, the expression of Occludin protein in Caco-2 cell monolayers in the control group was normal. In Caco-2 cell monolayers treated with 20 ng/ml TNF-α, the fluorescence signal of Occludin protein was weakened, the localization was abnormal, and the gap between cells was enlarged, which was rescued by ICA. DMSO had no significant effect on the expression of Occludin in Caco-2 cell monolayers.

To further confirm the protective effect of ICA on the depletion of Occludin induced by TNF-α in Caco-2 cells, protein and mRNA expressions of Occludin were detected by Western blot and qPCR, and the permeability of monolayers of Caco-2 cell was detected by 4-KD FITC-dextran and TEER. Results of WB and qPCR as shown in Figures 6D–F demonstrated that the expression of Occludin protein and mRNA in Caco-2 cell monolayers was markedly suppressed after TNF-α treatment. After the treatment of 5 μM ICA and 50 μM ICA, the reduced Occludin protein and mRNA expressions in TNF-α-induced Caco-2 cell monolayers were reversed. DMSO had no significant effect on the expressions of Occludin protein and mRNA in Caco-2 cell monolayers. As shown in Figures 6G,H, the permeability of Caco-2 cell monolayers to 4-KD FITC-dextran was low and the TEER value was high. However, the permeability of 4-KD FITC-dextran of Caco-2 cell monolayers was considerably increased, and the TEER value was impaired by the stimulation of TNF-α. After the intervention of 5 μM ICA and 50 μM ICA, the flux rate of TNF-α-induced Caco-2 cell monolayers to 4-KD FITC-dextran was distinctly reduced whereas the TEER value was increased. DMSO had no significant effect on the permeability of Caco-2 cell monolayers. Therefore, ICA rescued the elevated permeability of the monolayer barrier by alleviating TNF-α-induced Occludin damage to Caco-2 cells.

### ICA Reversed the Upregulation of miR-122a in Caco-2 Cell Monolayers Induced by TNF-α

**FIGURE 7:**
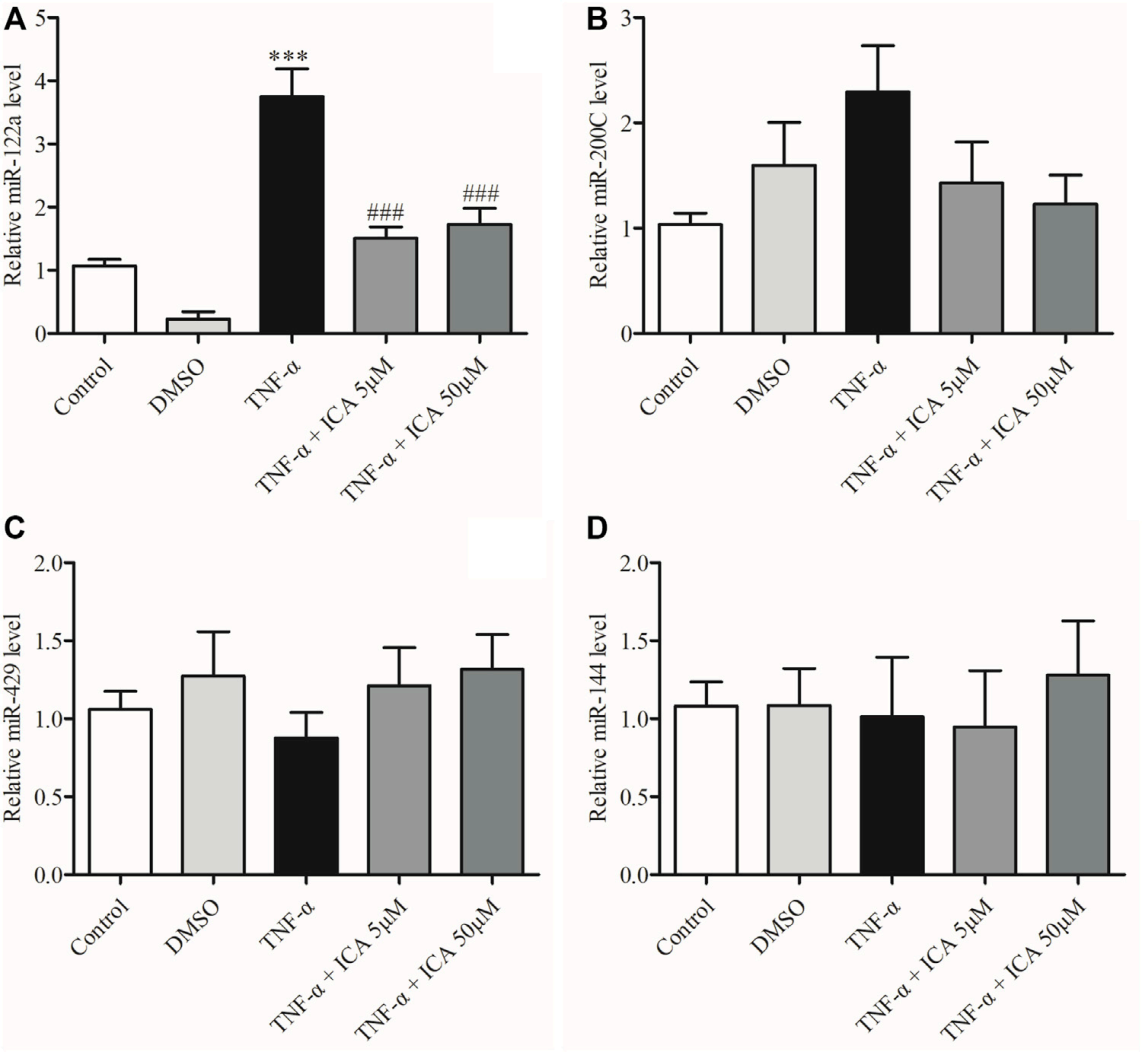
ICA reversed the upregulation of miR-122a in Caco-2 cells induced by TNF-α. Detected the expression levels of **(A)**miR-122a, **(B)**miR-200C, **(C)**miR-429, and **(D)**miR-144. ^***^ *p* < 0.001 vs. Control group. ^###^ *p* < 0.001 vs. TNF-α group, *n* = 5–12.

To further investigate the regulatory mechanism of ICA on the expression of Occludin, the expression levels of several miRNAs that could target Occludin mRNA in the intestinal epithelium were detected by qPCR, including miR-122a, miR-200c, miR-144, and miR-429. As shown in Figure 7A, the expression of miR-122a in Caco-2 cell monolayers was markedly increased by TNF-α treatment, which was significantly reversed by ICA. DMSO had no significant effect on the expression of miR-122a in Caco-2 cell monolayers. However, as shown in Figures 7B–D miR-200c, miR-144, and miR-429 miRNA unchanged obviously by either TNF-α or ICA. The results indicated that ICA relieved TNF-α-induced up-regulation of miR-122a expression.

### Overexpression of miR-122a Reversed the Protective Effect of ICA on Occludin Decrease and Increased Intestinal Permeability

**FIGURE 8:**
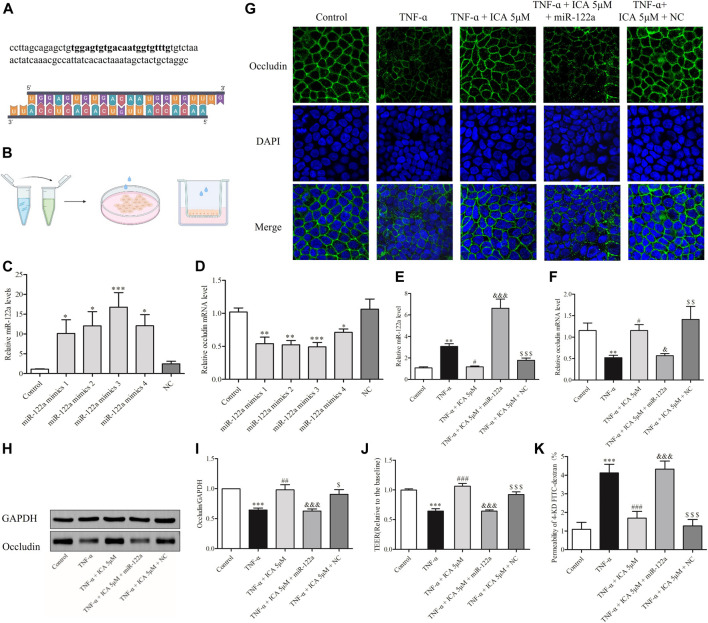
In Caco-2 cell monolayers, transfection of miR-122a mimics reversed the down-regulation protection of ICA against TNF-α -induced Occludin and induced improved permeability. **(A)** miR-122a mimics were designed based on the miR-122a gene. **(B)** Schematic diagram of transfection of miR-122a mimics in the Petri dish and Transwell Caco-2 cells. Effects of different transfection concentrations on the expression levels of **(C)** miR-122a and **(D)** Occludin mRNA in Caco-2 cells in the Petri dish. Changes in **(E)** miR-122a and **(F)** Occludin mRNA expression levels in cell monolayers. The miR-122a mimics group and the NC group were treated by transfecting miR-122a mimics or NC RNA for 24 h, and then treat with ICA and TNF-α. Changes in Occludin protein expression in Caco-2 cell monolayers were detected by **(G)** IF and **(H,I)** Western blot. Changes in permeability of Caco-2 cell monolayers were detected by **(J)** TEER and **(K)** 4-KD FITC-dextran. ^*^ *p* < 0.05 vs. Control group. ^**^ *p* < 0.05 vs. Control group. ^***^ *p* < 0.001 vs. Control group. ^#^ *p* < 0.05 vs. TNF-α group. ^##^ *p* < 0.01 vs. TNF-α group. ^###^ *p* < 0.001 vs. TNF-α group. ^&^ *p* < 0.05 vs. ICA 5 μM group. ^&&&^ *p* < 0.001 vs. ^$^ *p* < 0.05 vs. NC group. ^$$^ *p* < 0.05 vs. NC group. ^$$$^ *p* < 0.001 vs. NC group. miR-122a mimics 1: Lipofectamine 20001.5 μl, RNA 20 pmol; miR-122a mimics 2: Lipofectamine 2000 1.5 μl, RNA 40 pmol; miR-122a mimics 3: Lipofectamine 2000 2.5 μl, RNA 20 pmol; miR-122a mimics 4: Lipofectamine 2000 2.5 μl, RNA 40 pmol, *n* = 6–23.

To demonstrate that ICA alleviated TNF-α-induced impairment of Occludin and permeability in Caco-2 cell monolayers by reducing the expression of miR-122a, we transfected miR-122a mimics in Caco-2 cells. Figure 8A showed that miR-122a mimics were designed through genetic database screening. Transfection of miR-122a mimics in the Petri dish and Transwell Caco-2 cells showed in Figure 8B. As shown in Figures 8C,D, different concentrations of miR-122a mimics could increase the expression of miR-122a in cells and reduce the expression of Occludin mRNA. The efficient concentration ratio was selected to transfect Caco-2 cell monolayers. As shown in Figures 8E,F, consistent with the previous results, 5 μM ICA reversed the increase in TNF-α-induced miR-122a expression and impaired Occludin mRNA expression in Caco-2 cell monolayers. However, compared with the 5 μM ICA group, miR-122a mimics group showed visibly higher expression of miR-122a and lower expression of Occludin mRNA. There was no significant change in the NC group.

The expression of Occludin protein were shown in Figures 8G–I, which was similar to the mRNA expressions of Occludin. Compared with that in the normal group, the linear fluorescence of Occludin protein was broken after TNF-α treatment, which reappeared after ICA intervention. However, transfection of miR-122a mimics reversed the protective effect of ICA on TNF-α-induced Occludin down-regulation in Caco-2 cell monolayers. It was further tested whether transfection of miR-122a mimics could reverse the protective effect of ICA on the TNF-α-induced increase in the permeability of Caco-2 cell monolayers. As shown in Figures 8J,K, consistent with the previous results, TNF-α could increase the flux rate of 4-KD FITC-dextran and decrease the TEER value in Caco-2 cell monolayers. After treatment with ICA, the permeability of Caco-2 cell monolayers induced by TNF-α was markedly reversed. However, compared with those in the ICA group, the flux rate of 4-KD FITC-dextran was predominantly increased and the TEER value was reduced in the miR-122a-overexpressing group. DMSO had no significant effect on the permeability of Caco-2 cell monolayers. The results indicated that ICA alleviated TNF-α-induced Occludin disruption and intestinal barrier disruption by reducing the expression of miR-122a in Caco-2 cell monolayers.

## Discussion

Intestinal mucosal barrier function is essential for absorption of nutrients and maintenance of the homeostasis of the internal environment (Gehart and Clevers, 2019). Tight junction proteins including Occludin and Claudin are considered to be the most important mechanical barrier, and their reduction is key to the reduced intestinal barrier function in aging individuals (Zihni et al., 2016). Several studies have found that inflammatory cytokines lead to tight junction damaged and impaired intestinal barrier (Chen et al., 2015; Lee et al., 2015; Edogawa et al., 2020; He et al., 2020). ICA has been shown to have excellent protective effect against inflammation (Kong et al., 2015). Meanwhile, the recent study found the potential role of ICA in rescuing tight junction damage and protecting intestinal barrier function in piglets (Xiong et al., 2020). In this study, we found that ICA reduced the expressions of Occludin, Claudin1, and Claudin5 in colon tight junctions of aging rats, which were related to the reduction of inflammatory cytokines TNF-α and IL-1β and alleviation of colonic oxidative stress *in vivo*. And ICA attenuated TNF-α-induced Occludin disruption and epithelial barrier impairment by decreasing miR-122a expression in Caco-2 cell monolayers.

Inflammatory cytokines can markedly lead to the down-regulation of tight junctions (Buckley and Turner, 2018). TNF-α, among many inflammatory cytokines that destroy tight junctions, is one of the most concerned factors. In immune-mediated inflammatory bowel disease, TNF-α reduced the expression of Occludin protein to increase intestinal permeability and promoted the development of the disease (Su et al., 2013). IL-1β, like TNF-α, also apparently disrupts tight junctions, including Occludin, and Claudin1 (Wang et al., 2017). Aging leads to mild intestinal inflammation, such as increased expressions of inflammatory cytokines TNF-α and IL-1β, accompanied by loss of intestinal tight junctions and impaired intestinal barrier function (Ahmadi et al., 2020; Di et al., 2020). We investigated whether ICA had protective effects on tight junctions in rats with colonic inflammation. Consistent with previous results, the colon of aging rats had more inflammatory cell infiltration, and significantly increased TNF-α expression, as well as the expression of inflammatory cytokine IL-1β. Here we found that ICA reduced TNF-α and IL-1β expressions in the colon of aging rats, which indicating that ICA exerts anti-inflammatory effects. Moreover, ICA alleviated the loss of the expressions in Occludin, Claudin1 and Claudin5 in the colon of aging rats, which suggesting that ICA protected the loss of tight junctions to some degree. Meanwhile, we investigated the effects of ICA via TNF-α-stimulated Caco-2 cell monolayers and got similar results *in vitro*, demonstrating that ICA could improve the impaired tight junction barrier induced by inflammatory cytokines.

The occurrence and development of the inflammatory responses are closely related to oxidative stress. Oxidative stress is the imbalance of oxidation and antioxidant systems, resulting in the infiltration of inflammatory cells and tissue oxidative damage (Diaz de Barboza et al., 2017). Increased levels of colonic reactive oxygen free radicals and decreased antioxidant capacity are the primary mechanisms of inflammatory bowel disease (Tian et al., 2017). Meanwhile, oxidative stress occurs in the aging body’s eye, ovary, and gut (He et al., 2022; Ishaq et al., 2021; Cáceres-Vélez et al., 2022). Improving the oxidative stress state of the colon in mice effectively alleviates colon inflammation (Liu et al., 2019). These results suggest that oxidative stress may occur in the colon during the process of aging, leading to colonic inflammation and down-regulation of tight junction protein expression in intestinal epithelial cells. Meanwhile, our prediction results showed that ICA may alleviate colitis through multiple oxidative stress-related signaling pathways, such as AMPK, PI3K/AKT, HIF, Foxo4. Studies showed that increased AMPK could lead to the damage of intestinal tight junctions, such as ZO-1 and Occludin (Yang et al., 2017). Active PI3K/AKT could up-regulate the expression of Claudin2 and increase intestinal permeability (Yang et al., 2017). Up-regulated HIF-1α reduced the expression of Occludin, Claudin-1, and ZO-1 by unregulated miR-223 in rats and Caco-2 cells (Qu et al., 2022). In Foxo4-null mice, CD4^+^ T cell recruitment enhanced expressions of TNF-α and IFN-γ in colonic epithelium, accompanied by decreased expressions of ZO-1 and Occludin, and increased intestinal permeability (Zhou et al., 2009). Experiments in human colon-derived epithelial cell lines showed that ERK1/2 MAPK signaling pathway was activated during mitochondrial dysfunction, leading to increased IL-8 expression and intestinal barrier dysfunction, while the use of antioxidants reduced IL-8 expressions (Saxena et al., 2018). The above studies suggested that oxidative stress reduced the expression of tight junctions, especially by up-regulation of the expressions of inflammatory cytokines. And ICA alleviated the loss of tight junctions and the increased permeability in IPEC-J2 cells induced by *E. coli* through reducing oxidative stress, notably, the levels of inflammatory cytokines also significantly decreased (Wang et al., 2016). Those researches suggested that the effects of ICA on the lessening of colonic inflammatory cytokines s in aging rats may be related to the reduction of oxidative stress. Consistent with the findings of others, our results also showed that the activity of antioxidant enzymes and the expressions of antioxidant proteins in the colon of aging rats were lower than those of young rats. Thus, our results found that ICA relieved colonic oxidative stress in aging rats by increasing the expression of antioxidants, improving the antioxidant capacity of colon tissue and removing excess oxygen free radicals in the body.

Inflammatory cytokines can destroy Occludin and increase intestinal permeability by various mechanisms, including apoptosis, P38 MAPK, myosin light chain kinase, and protein kinase C (Yang et al., 2017), which mainly acts on the protein levels of Occludin, and the specific molecular mechanism is yet unclear. However, here the expressions of both the protein and mRNA of Occludin were obviously down-regulated by stimulation of TNF-α in Caco-2 monolayer cells. A large number of recent studies have shown that a variety of miRNAs directly target Occludin mRNA in the intestinal epithelium, resulting in attenuated Occludin expression and down-regulation of the intestinal barrier function. Studies on mouse intestinal epithelium and Caco-2 cells showed that TNF-α induced a significant increase in expression of miR-122a, which targets Occludin mRNA in the intestinal epithelium, leading to down-regulation of Occludin protein expression, and obvious permeability of inulin, meanwhile, inhibition of the expression of miRNA-122a can prevent the destructive effect of TNF-α (Ye et al., 2011). Several types of miRNAs in response to the stimulation of inflammatory cytokines have been reported to destabilize the mRNA of Occludin. In mouse intestinal epithelium and Caco-2 cells, miR-200C was up-regulated by IL-1β treatment and targeted to the 3’untranslated region of Occludin mRNA, resulting in reduced expression of Occludin (Rawat et al., 2020). MiR-429 and miR-144 were found to be increased in intestinal epithelium of diabetic mice and inflammatory bowel disease rats, respectively. These results indicate that miRNA plays an important role in the degradation of Occludin caused by TNF-α (Yu et al., 2016; Hou et al., 2017). While ICA could regulate the expression of several miRNAs, such as that ICA promoted osteogenic differentiation by inhibiting the expression of miRNA-34c (Liu et al., 2016), ICA attenuated the invasion and migration ability of cancer cells by inhibiting the expression of miRNA-625-3p (Fang et al., 2019). These studies suggest that ICA could downregulate the expressions of multiple miRNAs, but the mechanism is not clear at present. In this study, ICA was able to up-regulate both Occludin protein and mRNA levels, suggesting that ICA may protect the intestinal barrier by regulating miRNAs targeting Occludin mRNA. The experimental results showed that miR-122a was significantly up-regulated under the induction of TNF-α, and the treatment of ICA could reduce the expression of miR-122a. However, other miRNAs did not change visibly under the treatment of TNF-α or ICA, indicating that these miRNAs were not regulated by TNF-α or ICA. The above experimental results indicated that ICA reversed the impaired intestinal barrier function caused by TNF-α in a close association with down-regulation of miR-122a. To further verify the effects of ICA on the amelioration of TNF-α-induced intestinal barrier dysfunction by decreasing miR-122a expression, miR-122a was transfected into Caco-2 cells. It was found that the protective effect of ICA against TNF-α-induced degradation in both Occludin protein and mRNA in Caco-2 cell monolayers was abolished after transfection of miR-122a. Meanwhile, after transfection of miR-122a, the administration of ICA could not alleviate the decrease of TEER and the increase of 4-KD FITC-dextran flux rate caused by TNF-α treatment. These results showed that ICA could modify the loss of Occludin and the impaired intestinal barrier function in TNF-α-induced Caco-2 cells by down-regulating miR-122a. Recent studies found that Mitotically-associated long non-coding RNA reduces the transcription of miR-122a hepatocellular carcinoma (Zhang et al., 2019). As a nuclear transcription factor, NF-κB is activated by TNF-α and promotes the transcription of various tight junction-regulated genes, disrupting the intestinal barrier function (Luissint et al., 2016). Therefore, it is worth investigating whether ICA could regulate the expression of miR-122a through NF-κB, which we will further to explore the potential signaling pathway regulating miR-122a in the future.

However, here we didn’t determine the intestinal epithelial permeability and the expressions of miR-122a in the colonic epithelium of mice, which is a limitation of this study, and it is difficult to intervene and analyse the potential mechanism of damaged intestinal barrier function in the aging rats. And what caused the increased expression of miR-122a in the condition of inflammation is unknown and the mechanism is still deserved to be explored. Furthermore, to illustrate the universality of the protective effect of ICA on tight junctions, multiple kind of cell lines should be used. Our experiment does have the limitation of using only one kind of cell line.

## Conclusion

In summary, ICA attenuates the expressions of Occludin, Claudin1, and Claudin5 in colon, which were related to the reduction of inflammatory cytokines TNF-α and IL-1β and alleviation of colonic oxidative stress *in vivo*. And ICA alleviated TNF-α-induced Occludin disruption and epithelial barrier impairment by decreasing miR-122a expression in Caco-2 cell monolayers. Our study may provide new preventive and therapeutic ideas for diseases with intestinal inflammation-related disruption of intestinal barrier function.

## Data Availability

The original contributions presented in the study are included in the article/Supplementary Material, further inquiries can be directed to the corresponding authors.
